# New Appliances & Things Medical

**Published:** 1910-06-18

**Authors:** 


### NEW APPLIANCES & THINGS MEDICAL.

[We shall be glad to receive at our Office, 28 & 29 Southampton Street, Strand, London, W.C., from the manufacturers, specimens of all new preparations and appliances.]

### SURGICAL NEEDLES.

We have received from Messrs. W. Woodfield and Sons, of the Easemore Works, Redditch, various samples of their surgical needles. The firm supplies a sample card, which is of great interest and of practical value since it contains an assortment of all the varieties of surgical needles manufactured by Messrs. Woodfield. Here one finds Sims', Kelly's, Mayo's, Murphy's, Ferguson's, Martin's, Emmet's, and that very excellent hernia needle which is associated with the name of Hagedorn. Some of the varieties are extremely interesting, owing to the peculiar formation of the needles and the theoretical advantages which they are supposed to possess over ordinary needles. Every operator will doubtless have his own special pet among needles as among other surgeon's instruments, and will be able to defend his preferences against all the rest. Those, however, who desire, as it were, a bird's-eye view of the several varieties of needles on the market cannot do better than communicate with the above firm for one of their sample cards. The needles manufactured at Redditch are strong, of good temper, and, what is of infinitely greater importance, with excellent sharp points.

### GLOBE METAL POLISH.

Modern science has done much to assist the housewife in her labours, and the old-fashioned aid of "elbow-grease" is entirely out of place in a well-conducted household. Science has not, however, yet found a remedy to overcome the tarnishing effects of the atmosphere on bright metals. But it has made the polishing of metals a comparatively easy matter, if only the right material is used. This polish appears to be the ideal material. It has unique properties that not only enable it to produce a most brilliant shine with very little labour, but it preserves the metal from climatic action to a much larger extent than any other polish, with the result that the "Globe" shine is the most lasting. Taken in conjunction with the very little polish that it is necessary to use to obtain the brilliant shine one sees on metals polished with Globe, it makes it not only the easiest but the most economical metal polish one can buy. All grocers stock it, so that it is only a matter of stipulating "Globe" on the order to the grocer to obtain this—the best of metal polishes.

